# Adipocytokines and Inflammation in Patients and a Gerbil Model: Implications for Obesity-Related and Nonobese Diabetes

**DOI:** 10.1155/2024/9683512

**Published:** 2024-10-22

**Authors:** Hongjuan Fang, Xiaohong Li, Jianyi Lv, Xueyun Huo, Meng Guo, Xin Liu, Changlong Li, Zhenwen Chen, Xiaoyan Du

**Affiliations:** ^1^Department of Endocrinology, Aviation General Hospital, Beijing 100012, China; ^2^Department of Endocrinology, Beijing Tiantan Hospital, Capital Medical University, Beijing 100070, China; ^3^School of Basic Medical Science, Beijing Key Laboratory of Cancer Invasion and Metastasis Research, Capital Medical University, Beijing 100069, China; ^4^School of Basic Medical Science, Hebei University of Chinese Medicine, Shijiazhuang 050200, China; ^5^Laboratory for Clinical Medicine, Capital Medical University, Beijing 100069, China

**Keywords:** adipose inflammation, ectopic fat deposition, lean diabetes, obese diabetes

## Abstract

**Background:** Obesity is a predisposing risk factor for type 2 diabetes mellitus (T2DM). Actually, not only obese/overweight but also nonobese/lean individuals may be prone to T2DM. This study is aimed at identifying the contribution of adipose tissue to the development of nonobese diabetes (NOD) and obese diabetes (OD).

**Methods:** Serum samples from the nonobese nondiabetes (NOND, *n* = 47, age = 46.8 ± 8.4, BMI ≤ 23.9 kg/m^2^) controls, NOD (*n* = 48, age = 50.7 ± 6.5, BMI ≤ 23.9 kg/m^2^) and OD (n = 65, *age* = 49.8 ± 10.2, BMI ≥ 28 kg/m^2^) patients were utilized to measure the expression of metabolic indicators, adipocytokines, inflammatory factors. Different adipose depots from offspring with corresponding blood glucose and obesity levels of a spontaneously diabetic gerbil line with various degrees of diabetic penetrance and body weights were examined for adipocytokines and inflammation factors detected by ELISA and western blot. Adipose tissue volume and fat cell size of the gerbils were evaluated by magnetic resonance imaging and immunohistochemistry, respectively.

**Results:** The study yielded four key findings. Firstly, in comparison to the NOD group, the OD group exhibited more severe insulin resistance (IR) and metabolic dysfunction in both patients and gerbils, attributed to higher visceral adipose tissue mass and larger fat cell sizes. Secondly, in gerbils, gonadal fat deposition was linked to obesity development, whereas kidney fat deposition correlated with obesity and diabetes occurrence. Thirdly, in both patients and gerbils, the interplay between adiponectin and leptin levels in serum may significantly influence the development of obesity and diabetes. Lastly, heightened expression of MCP3 in gerbils' kidney adipose tissue may serve as a pivotal factor in initiating obesity-associated diabetes.

**Conclusions:** Our study, which may be considered a pilot investigation, suggests that the interaction of adipocytokines and inflammation factors in different adipose depots could play diverse roles in the development of diabetes or obesity.

## 1. Background

Type 2 diabetes mellitus (T2DM), one of the largest chronic health problems worldwide, is closely intertwined with the increasing incidence of obesity [1]. It is estimated that 60%–90% of T2DM patients are obese [2]. Numerous studies confirmed that obesity–induced insulin resistance (IR) accelerates pancreatic islet exhaustion, contributing to the onset of T2DM [3, 4]. In the real world, not all obese individuals develop T2DM, and diabetes with low or normal body mass index (BMI) is not rare [5]. Individuals with nonobese or obese developed diabetes may be through different pathophysiological mechanisms [6, 7]. Due to the scarcity of spontaneous nonobese diabetic (NOD) animal models [8], the precise etiology of hyperglycemia in patients with low or normal BMI remains to be fully elucidated.

Adipose tissue, the largest organ in the human body, produces multiple adipocytokines and inflammatory factors [9]. Dysfunction of adipose tissue is a central driver for obesity-associated diabetes [10, 11]. Emerging findings indicate that variations in fat deposition in the liver or in the pancreas may significantly influence the risk of diabetes, particularly in nonobese individuals [12, 13]. Understanding definitive discrepancies in the profiles and function of adipose tissue between obese diabetes (OD) and NOD patients is crucial to unravel the pathophysiology of both conditions. While considerable research has focused on characterizing OD patients [14–16], relatively little is known about NOD [17, 18].

In this study, we investigated the clinical characteristics of patients with OD and NOD, including metabolic indicators, adipocytokines, and inflammatory factors. However, analyzing the distribution and function of adipose tissue presents a challenge due to difficulties in collecting fat from the adipose tissues of patients. Fortunately, while establishing a spontaneously diabetic gerbil line, we identified four distinct phenotypes: nonobese nondiabetic (NOND), obese nondiabetic (OND), NOD, and OD gerbils [19]. Utilizing this spontaneous diabetic gerbil model, we explored the definitive distinctions in adipose tissue function between OD and NOD.

## 2. Materials and Methods

### 2.1. Subjects

Human blood samples were collected from the Department of Endocrinology, the Chinese PLA General Hospital. Adipocytokines and inflammatory factors were measured in duplicate using the ELISA method with commercially available kits. All participants gave their written informed consents, and the study was approved by the Institutional Ethical Review Board of Chinese PLA General Hospital (No. S2016-147-03KY2013-015-02). Forty-seven NOND controls (20 females, 42.5%; 27 males, 57.5%; mean age = 46.8 ± 8.4 years; mean BMI = 21.65 ± 1.81 kg/m^2^), 48 NOD patients (25 females, 52.1%; 23 males, 47.9%; mean age = 50.7 ± 6.5 years; mean BMI = 22.68 ± 1.24 kg/m^2^), and 65 OD patients (27 females, 41.5%; 38 males, 58.5%; mean age = 49.8 ± 10.2 years; mean BMI = 30.71 ± 2.25 kg/m^2^) were included. We discarded the OND group, as only one human blood sample was collected. T2DM was defined as a history of T2DM, or the use of insulin and/or oral hypoglycemic agents, excluding T1DM and other types of DM.

### 2.2. Animals

The animal study was reviewed and approved by the Ethics Committee of Capital Medical University (No. AEEI-2017-032), and the experimental protocols adhered to the guidelines outlined in the National Institutes of Health Guide for the Care and Use of Laboratory Animals. One-year-old Mongolian gerbils, bred with free access to food and water, were housed in the animal facility of the Capital Medical University under standard laboratory conditions at 20°C–24°C with a 12-h light/dark cycle and 50%–70% relative humidity. Body weight (BW) was measured weekly using an electronic scale (Torrey, City of Mexico, State of MEX, Mexico).

According to the weight range of adult gerbils, adult gerbils weighing between 60 and 75 g are categorized as nonobese, whereas those weighing ≥ 94 g are classified as obese. Based on BW and results from intraperitoneal glucose tolerance test (IPGTT), Mongolian gerbils were classified into four groups: NOND (*n* = 10, BW = 64.0 ± 3.1 g, with fasting glucose (FPG) < 5.2 mmol/L and 2 h glucose tolerance (PG2h) < 6.8 mmol/L), OND (*n* = 5, BW = 100.6 ± 4.4 g, FPG < 5.2 mmol/L and PG2h < 6.8 mmol/L), NOD (*n* = 5, BW = 66.8 ± 3.5 g, FPG ≥ 5.2 mmol/L and PG2h ≥ 6.8 mmol/L), and OD (*n* = 12, BW = 101.2 ± 4.7 g, FPG ≥ 5.2 mmol/L and PG2h ≥ 6.8 mmol/L).

### 2.3. Metabolism Indicators, Adipocytokines, and Inflammation Factors

For gerbils, the IPGTT was conducted via intragastric administration with a glucose intake (2 g/kg) following a 16-h fasting period, and insulin tolerance test (ITT) was performed by intraperitoneal administration of insulin (0.75 U/kg, Novo Nordisk, China) after a 4-h fasting, as previously described [19]. Whole blood samples were collected from the gerbils' orbital sinuses into tubes and serum was separated and frozen at −80°C for ELISA testing. The ELISA kits of insulin (Millipore, Germany), adiponectin (Abcam, United States), and leptin (R&D, United States) were used to measure serum levels of insulin, adiponectin, and leptin in humans and gerbils. Other cytokines of humans and gerbils including IL-13, IL-1*β*, IL-2, IL-10, IL-22, IL-23, IL-27, IP-10, MCP-3, MIP-1*α*, RANTES, eotaxin, ENA-78, IL-6, TNF-*α*, MCP-1, G-CSF, Galectin-3, IFN-*γ*, IL-28, and VEGF-A were analyzed using ProcartaPlex multiplexing immunoassay kits (eBioscience, United States) on a Luminex 200 instrument (Luminex, United States). Results were analyzed using ProcartaPlex Analyst 1.0 software.

### 2.4. Measurement of Adipose Tissue Volume and Weight

All Mongolian gerbils were fasted overnight (12 h) and then anesthetized with an intraperitoneal injection of 3% pentobarbital sodium (30 mg/kg). Fat tissue volume was assessed using Bruker's PharmaScan 7T MRI Scanner (Bruker, Germany) with a multislice multiecho sequence in vivo. Image parameters were set as follows: repetition time, 500.000 ms; echo time, 8.000 ms; number of averages, 5; slice thickness, 1 mm; matrix size, 256 × 256. Various adipose depots (subcutaneous fat, gonad fat, kidney fat, pancreatic fat, and mesenteric fat) were collected and weighed.

### 2.5. Measurement of Fat Cell Size

The adipose tissue of gerbils was fixed in 4% paraformaldehyde for 2 weeks. Following paraffin embedding, 2-*μ*M thick slices were cut and placed on glass slides. Paraffin sections were stained with hematoxylin and eosin (H&E) and then microscopically examined, and fat cell areas were analyzed using ImageJ software (National Institutes of Health, United States).

### 2.6. Western Blot Analysis

Proteins were extracted from subcutaneous, gonadal, and kidney fat tissues of gerbils using the protein extraction kit (CWBIO, China). Subsequently, the proteins were separated via sodium dodecyl sulfate-polyacrylamide gel electrophoresis and transferred to polyvinylidene difluoride membranes. The membranes were then blocked in Tris-buffered saline with 0.1% Tween containing 5% skimmed milk for 1 h at room temperature, followed by overnight incubation at 4°C with primary antibodies against adiponectin (Abcam, 1:1000), leptin, MIP-1*α*, MCP-3 (all from R&D, 1:1000), and *β*-actin (Cell Signaling Technology, 1: 1000). Subsequent to primary antibody incubation, membranes were incubated with an isotype-matched horseradish peroxidase-labeled anti-IgG antibody (1:1000) for 1 h, and the blots were developed using chemiluminescence reagent (Advansta, Menlo Park, CA, United States). The bands were visualized and quantified using ImageJ software v1.49.

### 2.7. Statistical Analysis

Statistical analyses were conducted using SPSS 21.0 (SPSS Inc., United States). All values were presented as means ± S.D. of independent experiments. Comparisons among more than two groups were performed using one-way analysis of variance. Spearman's tests were used for correlations involving nonparametric variables. Statistical significance was defined as *p* < 0.05.

## 3. Results

### 3.1. Adipocytokines and Inflammation in Patients With OD and NOD

**Figure 1:**
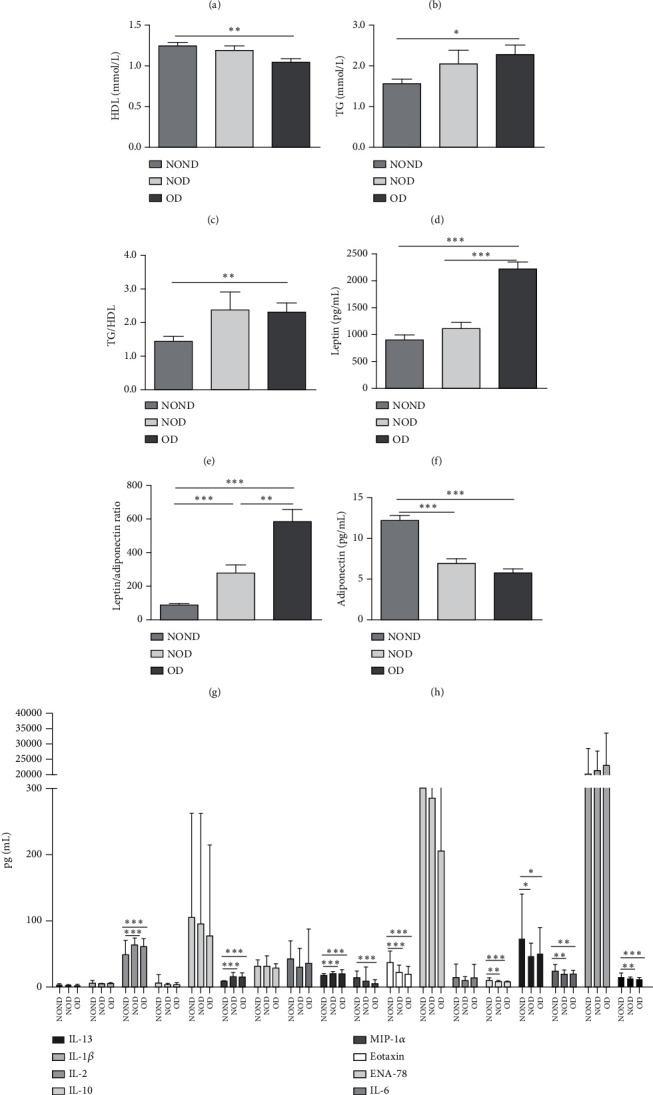
Expression of metabolic indicators, adipocytokines, and inflammation factors in NOND controls as well as NOD and OD patients. (a, b) The levels of fasting insulin and postprandial insulin were detected by ITT. (c–e) The level of HDL, TG, and TG/HDL ratio in NOND (*n* = 47) controls as well as NOD (*n* = 48) and OD (*n* = 65) patients. (f–h) Serum levels of adipocytokines and (i) inflammatory factors were measured using the ELISA method. *Notes:*⁣^∗^*p* < 0.05, ⁣^∗∗^*p* < 0.01, and ⁣^∗∗∗^*p* < 0.001 showed significant differences between the groups.

In humans, fasting insulin and 2-h insulin levels after sugar load were significantly higher in NOD and OD patients compared to NOND patients (Figures 1(a) and 1(b)). The triglyceride (TG)/high-density lipoprotein (HDL) ratio was shown to be an excellent predictor of IR, even in individuals of normal weight [20]. HDL levels were lower, while TG levels were higher in NOD and OD patients compared to NOND patients (Figures 1(c) and 1(d)). Furthermore, the TG/HDL ratios were higher in OD and NOD patients than in NOND patients (Figure 1(e)).

Adiponectin and leptin are two adipocytokines secreted by white adipose tissue, and the elevated leptin/adiponectin ratio (LAR) has been confirmed as a biomarker of adipose tissue dysfunction [21]. The results demonstrated an increasing trend in serum leptin levels and LAR, while serum adiponectin levels exhibited a decreasing trend in the NOND, NOD, and OD groups. LAR was higher in the OD group compared to the NOD group (Figures 1(f), 1(g), and 1(h)). Serum levels of IL-2, IL-23, and MCP-3 were higher in the obese or diabetic groups than in the NOND group. Furthermore, serum levels of MIP-1a, TNF-a, G-CSF, and IFN*γ* were lower in the OD group compared to the NOD group (Figure 1(i)).

### 3.2. The Metabolic Profile of OD and NOD Gerbils

**Figure 2:**
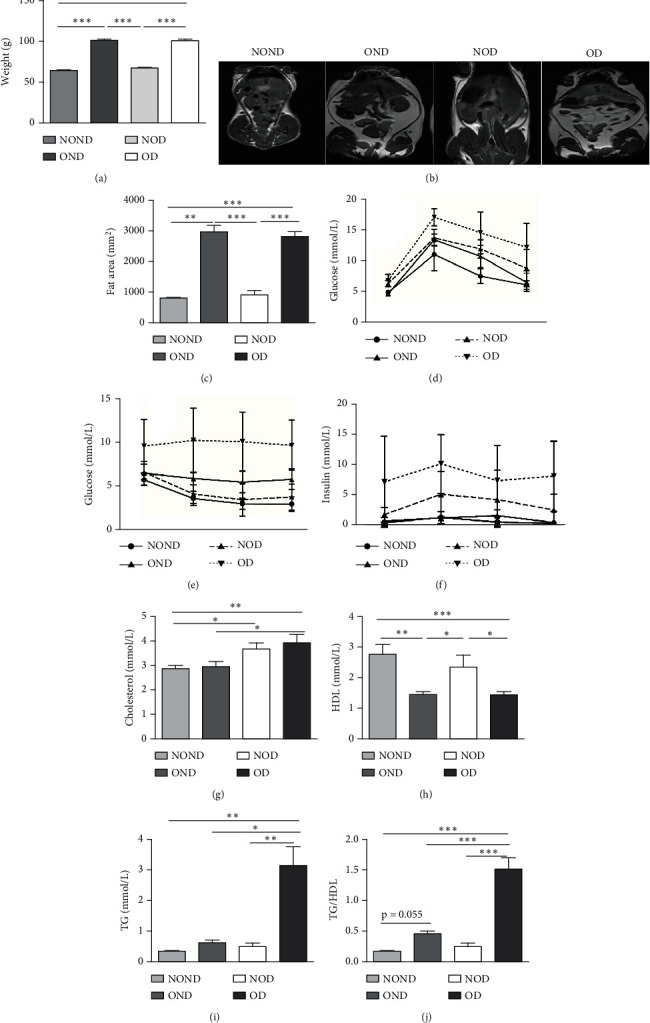
The metabolic profile of obese and nonobese diabetic gerbils. (a) The body weights of the gerbils. (b, c) The fat areas were detected by magnetic resonance detection. (d–f) The glucose tolerance, insulin sensitivity, and insulin resistance were measured by IPGTT and ITT. (g–j) The level of cholesterol, HDL, TG, and TG/HDL ratio in NOND (*n* = 10), OND (*n* = 5), NOD (*n* = 5), and OD (*n* = 12) groups. Notes: ⁣^∗^*p* < 0.05, ⁣^∗∗^*p* < 0.01, and ⁣^∗∗∗^*p* < 0.001 showed significant differences between the groups.

The BWs of gerbils were significantly higher in obese groups than in nonobese groups (Figure 2(a)). MRI detection revealed that the fat areas of the OND and OD gerbils were significantly greater than those of the NOND and NOD groups (Figures 2(b) and 2(c)).

Regardless of obesity status, IGPTT indicated impaired glucose tolerance in diabetic (NOD and OD) groups (Figure 2(d)). Irrespective of diabetes status, IGPTT demonstrated a decrease in insulin sensitivity in the obese (OND and OD) groups (Figure 2(e)). The ITT showed higher insulin levels in the diabetic (NOD and OD) groups compared to the nondiabetic (NOND and OND) groups (Figure 2(f)). Regardless of obesity status, the gerbils exhibited higher cholesterol levels in the diabetes (NOD and OD) groups compared to the nondiabetes (OND and NOND) groups (Figure 2(g)). Regardless of diabetes status, the gerbils exhibited lower HDL levels in the obese (OND and OD) groups compared to the nonobese (NOND and NOD) groups (Figure 2(h)). The TG level and TG/HDL ratio were higher in the OD group than in the NOD and OND groups (Figures 2(i) and 2(j)).

In comparison to the NOND group, the OND group exhibited normal glucose metabolism, increased insulin requirements, and IR. The NOD group showed glucose intolerance and increased insulin requirements, but normal insulin sensitivity. However, the OD group was characterized by glucose intolerance, increased insulin requirements, and decreased insulin sensitivity. These findings indicated that the metabolic profile of OD and NOD gerbils closely resemble those of humans, rendering them an ideal animal model for investigating the mechanisms underlying OD and NOD.

### 3.3. Adipocytokines and Inflammation in OD and NOD Gerbils

**Figure 3:**
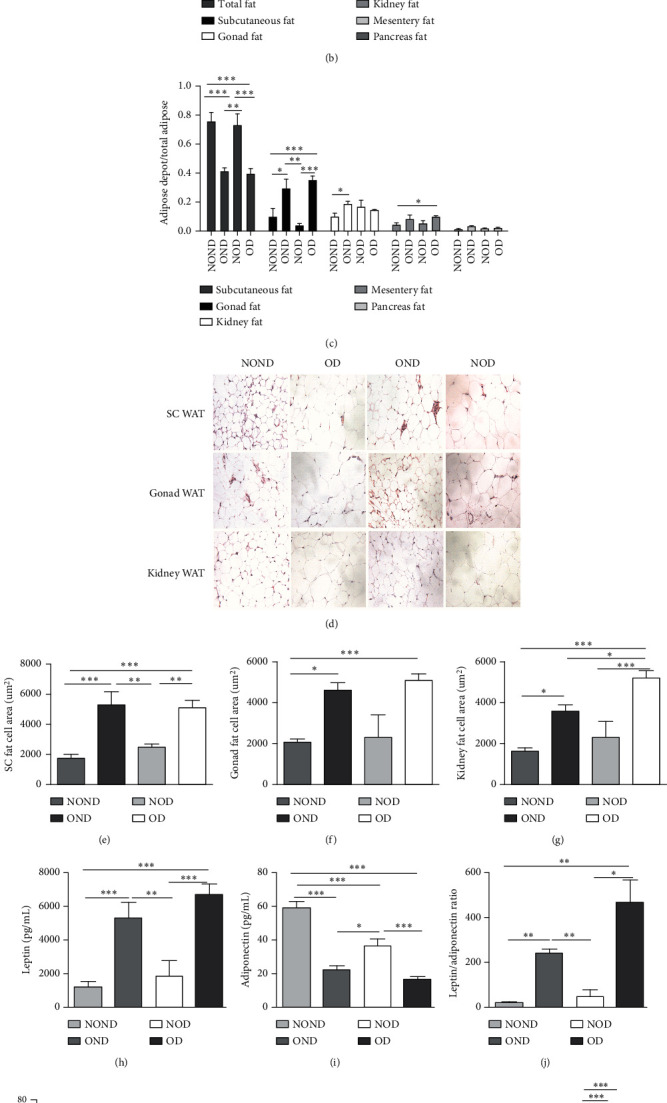
The levels of adipocytokines and inflammation in obese and nonobese diabetic gerbils. (a) The mass of adipose tissue of different depots including subcutaneous, gonad, kidney, mesentery, and pancreas fat and (b) the relative weight based on the total body weight or (c) total adipose mass were measured and calculated. (d–g) The fat cell sizes of subcutaneous, gonad, and kidney tissue were measured by HE staining. (h–j) Serum levels of adipocytokines and (k) inflammatory factors were measured using the ELISA method. Groups were set up the same as in Figure 2. *Notes:*⁣^∗^*p* < 0.05, ⁣^∗∗^*p* < 0.01, and ⁣^∗∗∗^*p* < 0.001 showed significant differences between the groups.

OND and OD gerbils exhibited greater subcutaneous adipose tissue (SAT) mass and visceral adipose tissue (VAT) mass compared to NOND and NOD gerbils (Figure 3(a)). Compared to NOD and NOND gerbils, OND and OD gerbils exhibited a higher subcutaneous fat to BW, while a lower subcutaneous fat to total adipose ratio (Figures 3(b) and 3(c)). This suggests that the contribution of subcutaneous fat to total fat mass decreases significantly in obese individuals.

The ratios of VAT (including gonadal, kidney, mesenteric, and pancreatic fat) to BW and total adipose weight were notably higher in the obese groups compared to the nonobese groups. Specifically, the ratios of gonadal adipose weight to total adipose weight were elevated in the obesity (OND and OD) groups relative to the nonobese (NOND and NOD) groups. The ratios of kidney adipose weight to total adipose weight were higher in the obese or diabetic (NOD, OND, and OD) groups compared to the NOND group (Figures 3(b) and 3(c)). These findings suggest a distinct association between gonadal adipose tissue and the development of obesity, while kidney adipose tissue appears to be linked to the onset of obesity and diabetes.

The OD group exhibited larger fat cell sizes and greater fat mass in subcutaneous, gonadal, and kidney adipose tissues compared to the NOD group. However, no significant differences were found between the NOD gerbils and the NOND group. In contrast, while no significant differences in fat cell sizes were observed between the OD and OND groups in subcutaneous and gonadal adipose tissues, larger fat cell sizes were specifically noted in kidney adipose tissues of the OD group (Figures 3(d), 3(e), 3(f), and 3(g)).

The expression of adipocytokine levels exhibited a high degree of similarity between gerbils and humans. Regardless of diabetes status, the obese group exhibited higher levels of leptin, lower levels of adiponectin, and higher LAR compared to the nonobese group (Figures 3(h), 3(i), and 3(j)). OD gerbils demonstrated impaired adipose tissue function, increased ectopic fat deposition, and reduced insulin sensitivity compared to NOD gerbils, emphasizing the central role of adipose tissue function in the development of diabetes.

Regardless of diabetes status, the levels of IL-2 increased, while IL-28 decreased in the OND and OD groups. Moreover, the IL-22 and IL-23 levels were highest in the NOD group. Additionally, IP-10, MIP-1a, and MCP-3 levels exhibited an increase in OD gerbils (Figure 3(k)). These observations imply that inflammation is intricately linked to the development of both obesity and diabetes. Furthermore, the potential pathogenesis underlying obesity and T2DM may be attributed to the activation of inflammatory responses by adipocytokines.

### 3.4. Correlation Analyses of Adipocytokines and Inflammation

Based on the expression of serum adipocytokines, metabolic indicators, and inflammation factors across different groups of humans and gerbils, we analyzed the correlations between adipocytokines (leptin and adiponectin) and differentially expressed metabolic indicators and inflammation factors.

In humans, compared to the NOND group, the diabetes (obese or nonobese) groups exhibited negative correlations between adiponectin and IL-2 and IL-23, while showing positive correlations with ENA78, eotaxin, and IP-10, along with positive correlations between leptin and insulin and IL-23. Furthermore, compared to the NOD group, the OD group displayed a negative correlation between adiponectin and TG, while positive correlations with IL-13, IL-2, and MCP-3, along with positive correlations between leptin and IL-6 and MIP1*α* (Table 1).

In gerbils, we investigate the correlation between adipocytokines and inflammation across four pairs: NOND-OND, NOND-NOD, NOND-OD, OND-OD, and NOD-OD. Compared to the NOND group, the OND, NOD, and OD groups exhibited negative correlations between adiponectin with IL-10 and IL-27. Additionally, compared to the NOD group, the OD group displayed a negative correlation between adiponectin and leptin, insulin, IL-13, and IP-10, while showing positive correlations with HDL and IL-22, along with positive correlations between leptin and eotaxin, IL-13, IL-2, and IP-10 (Table 2).

However, upon analyzing correlations among nondiabetic individuals with or without obesity (NOND-OND), diabetic individuals with or without obesity (NOD-OD), and obese diabetics (NOND-OD), the findings align with those observed in gerbils, only negative correlations between adiponectin and leptin, and positive correlations between adiponectin and HDL.

### 3.5. Cytokine Levels in Different Adipose Depots

Based on the serum cytokine levels of different gerbil groups, we assessed the protein expression of leptin, adiponectin, MCP-3 and MIP-1*α* in the subcutaneous, gonadal, and kidney adipose tissues of gerbils.

**Figure 4:**
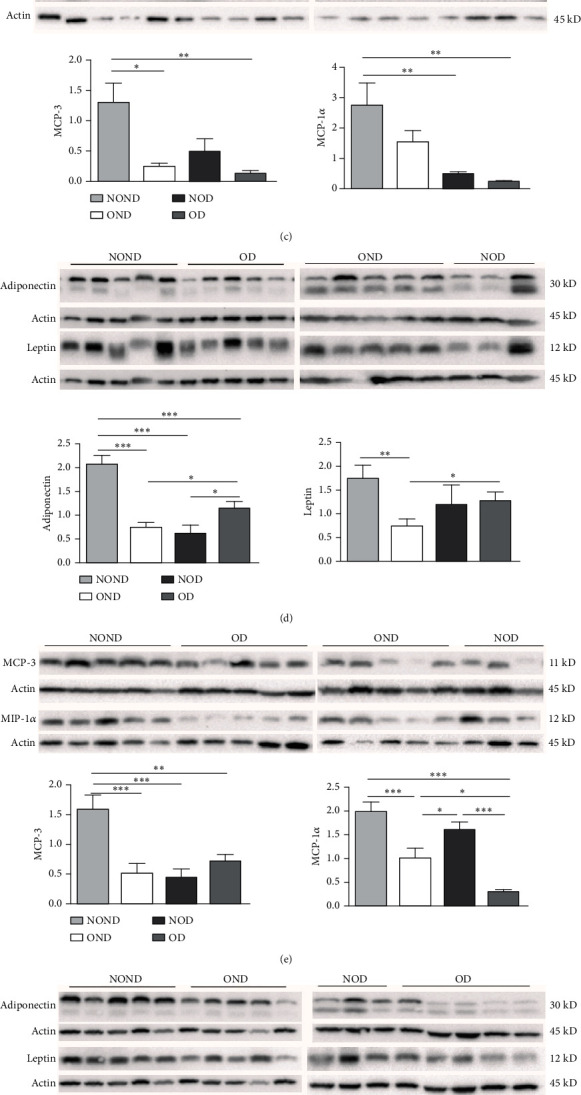
The levels of adipocytokines and inflammation factors in different adipose depots. The expression of MCP-3, MIP-1*α*, adiponectin, and leptin in the (a, b) subcutaneous, (c, d) gonadal, and (e, f) kidney adipose were detected by Western blot. *N* = 5 for each group for each protein except *n* = 4 for the OND and NOD groups for MCP-3, MIP-1*α*, adiponectin, and leptin. (g, h) The expression levels of MCP-3, MIP-1*α*, adiponectin, and leptin in different adipose depots of the same group. *Notes:*⁣^∗^*p* < 0.05, ⁣^∗∗^*p* < 0.01, and ⁣^∗∗∗^*p* < 0.001 showed significant differences between different adipose depots.

In SAT, regardless of diabetes status, the expression of MCP-3 and MIP-1*α* levels was reduced in the obesity (OD and OND) groups. The expression of leptin and adiponectin levels in the OD group was the lowest among the four groups (Figures 4(a) and 4(b)).

In gonadal adipose tissue, the expression of MCP-3 and MIP-1*α* decreased in the obese or diabetic groups (OND, NOD, and OD). The expression of adiponectin was higher in the OD group than in the OND and NOD groups, and the expression of leptin in the OND group was the lowest among the four groups (Figures 4(c) and 4(d)).

In kidney adipose tissue, the expression of MCP-3 and MIP-1*α* were significantly higher in NOND than in the obese or diabetic groups (OND, NOD, and OD), and the expression of MIP1*α* in the OD group was the lowest among the four groups. The levels of leptin and adiponectin were significantly decreased in obese groups, regardless of diabetes status (Figures 4(e) and 4(f)).

We investigated the expression of the same cytokine in different adipose depots (Figures 4(g) and 4(h)). In obese groups (OND and OD), MCP3 expression was significantly higher in kidney adipose tissue than in subcutaneous and gonadal adipose tissue. The expression of MIP1*α* in all adipose depots of the OD group was the lowest. These findings suggest that the expression of MCP-3 in kidney adipose depots may play a vital role in the development of diabetes or obesity (OND and OD), while having less effect on NOD.

In both the OND and NOD groups, the expression of leptin and adiponectin was higher in SAT compared to gonadal and kidney adipose tissue. However, the expression of leptin and adiponectin in kidney adipose depots of the OD group was the lowest. The disproportionate expression of adiponectin and leptin in adipose tissue seemed to have little association.

## 4. Discussion

To investigate the contribution of adipose tissue to the development of OD and NOD, especially in relation to metabolic dysfunction and inflammation, we measured the levels of metabolic indicators, adipocytokines, and inflammatory cytokines in the sera of diabetic patients with and without obesity. Using a spontaneously diabetic gerbil model with various degrees of diabetic penetrance and BWs, we verified the patients' results in both the gerbils' sera and their adipose depots. We compared the parameters among different groups of patients and gerbils, with or without diabetes and/or obesity, and analyzed interactions and correlations among these parameters. Our results indicated that the OD group exhibited more severe IR and metabolic dysfunction than the NOD group in both patients and gerbils. Alterations in adiponectin and leptin levels, and their interactions, may significantly influence the development of obesity and diabetes. In gerbils, gonadal fat deposition was associated with the development of obesity, while kidney fat deposition correlated with the occurrence of both obesity and diabetes, possibly driven by higher levels of MCP3 expression.

In our study, we observed elevated adipose tissue mass, including various regional fat content, adipose mass/BW ratio, and adipose depot/total fat ratio, in obese groups (OND and OD gerbils) compared to nonobese groups (NOD and NOND gerbils). The higher mass of adipose tissue in obesity groups (diabetes or not) may be a result of the larger fat cell sizes in different adipose depots. Additionally, cytokines in different adipose depots are more closely associated with obesity or OD than those with NOD. We propose the pathogenic mechanisms underlying NOD appear distinct from those of OD, particularly concerning adipose storage and functionality.

Accumulating evidence suggests a direct correlation between variations in the body fat distribution, adipokine secretion, and T2DM [22, 23]. However, due to ethical considerations, it is challenging to collect fat samples from various parts of patients for studying fat content levels and cytokines expression. Our study reveals that OD and OND gerbils display identical adipose distribution and metabolic profiles to those observed in humans. These findings suggest that the spontaneously diabetic gerbil line could offer a valuable model for understanding the role of adipose depots in obesity or diabetes development.

Numerous studies have found that the accumulation of SAT may defend against T2DM risk, while VAT is associated with adverse metabolic effects [24, 25]. Our findings reveal that OD gerbils exhibited significant deposition of VAT, a higher ratio of VAT, and larger fat cell size. However, compared to OD gerbils, NOD gerbils showed no significant difference in SAT mass or the ratio of SAT/BW, indicating a weak correlation between SAT and NOD. Our findings are consistent with the notion that obesity and T2DM are associated with abnormally enlarged adipocytes and excess lipid accumulation [26].

A longitudinal study found that the hepatic fat fraction accumulation was the only depot to significantly predict clinical markers of type 2 diabetes risk, fasting glucose, and HbA1c [27]. A recently published Mendelian study also found that fat in the liver plays a crucial role in determining whether people with obesity are likely to develop type 2 diabetes or not [28]. Our study revealed a clear association between gonadal adipose tissue and obesity development, while kidney adipose tissue seems to be related to the onset of obesity and diabetes. The expression of MCP-3 in kidney adipose depots may play a crucial role in the development of diabetes or obesity (OND and OD), with a lesser impact on NOD.

Previous studies have shown connections between adipocytokines and inflammatory factors in individuals with T2DM or obesity [29, 30]. Our research has uncovered that the distribution of ectopic fat depots and the expression of adipocytokines, alongside inflammatory reactions in various adipose depots, may have varying impacts on the onset of diabetes or obesity. The interplay of adiponectin and leptin in serum is closely related to the development of obesity or obesity-related disorders but appears to have minimal association with adipose tissue.

We found a high degree of similarity or identicality in the metabolic profiles, adipocytokine levels, and adipose distribution in patients and corresponding gerbil groups. Therefore, we used the adipose depots of gerbils as a model to study the effects of human fat tissues on the development of obesity and diabetes. However, please note that differences exist in the morphology of adipose tissues between gerbils and humans, such as variations in lipid droplet size and number. Thus, we must interpret our results from gerbils to humans with caution.

In conclusion, our results implied that the characteristics of fat mass, body fat distribution, and adipose tissue dysfunction are pivotal factors in the development of obesity-related diabetes. Although our study provides detailed information on the profiles and function of adipose tissue in OD and NOD, it is important to note that this is an early, pilot study, and drawing statistically robust conclusions will require the investigation of significantly larger cohorts. The intricate interactions among adipocyte dysfunction, obesity, and diabetes underscore the therapeutic potential of adipose tissue in addressing both obesity and diabetes.

## Figures and Tables

**Table 1:** Correlation analyses of metabolic indicators, adipocytokines, and inflammation factors in human serum of NOND, NOD, and OD groups (*n* = 47, 48, and 65, respectively).

	**NOND-NOD**	**NOND-OD**	**NOD-OD**
Adiponectin-leptin	—	−0.27⁣^∗∗^	—
Adiponectin-cholesterol	—	—	—
Adiponectin-HDL	—	0.32⁣^∗∗∗^	—
Adiponectin-TG	—	−0.33⁣^∗∗∗^	−0.25⁣^∗∗^
Adiponectin-insulin	—	−0.41⁣^∗∗∗^	—
Adiponectin-RANTES	—	—	—
Adiponectin-ENA78	0.21⁣^∗^	0.23⁣^∗^	—
Adiponectin-eotaxin	0.33⁣^∗∗∗^	0.39⁣^∗∗∗^	—
Adiponectin-IL13	—	—	0.23⁣^∗^
Adiponectin-IL2	−0.25⁣^∗^	−0.26⁣^∗∗^	0.31⁣^∗∗∗^
Adiponectin-IL10	—	—	—
Adiponectin-IL22	—	0.30⁣^∗∗∗^	—
Adiponectin-IL23	−0.39⁣^∗∗∗^	−0.46⁣^∗∗∗^	—
Adiponectin-IL27	—	—	—
Adiponectin-IP10	0.29⁣^∗∗^	0.29⁣^∗∗^	—
Adiponectin-MCP3	—	—	0.29⁣^∗∗^
Adiponectin-MIP1*α*	—	0.28⁣^∗∗^	—
Leptin-cholesterol	—	—	—
Leptin-HDL	—	−0.37⁣^∗∗∗^	—
Leptin-TG	—	0.23⁣^∗^	—
Leptin-insulin	0.51⁣^∗∗∗^	0.65⁣^∗∗∗^	—
Leptin-ENA78	—	−0.22⁣^∗^	—
Leptin-eotaxin	—	−0.21⁣^∗^	—
Leptin-GCSF	—	−0.23⁣^∗^	—
Leptin-IFN*γ*	—	−0.25⁣^∗∗^	—
Leptin-IL13	—	—	—
Leptin-IL2	—	0.41⁣^∗∗∗^	—
Leptin-IL23	0.21⁣^∗^	0.57⁣^∗∗∗^	—
Leptin-IL6	—	—	0.20⁣^∗^
Leptin-IP10	—	—	—
Leptin-MCP3	—	0.34⁣^∗∗∗^	—
Leptin-MIP1*α*	—	−0.27⁣^∗∗^	0.22⁣^∗^
Leptin-TNF*α*	—	−0.34⁣^∗∗∗^	—

*Note:* “—” means no correlation was found.

⁣^∗^*p* < 0.05.

⁣^∗∗^*p* < 0.01.

⁣^∗∗∗^*p* < 0.001.

**Table 2:** Correlation analyses of metabolism indicators, adipocytokines, and inflammation factors in gerbil serum of NOND, OND, NOD, and OD groups (*n* = 10, 5, 5, and 12, respectively).

	**NOND-OND**	**NOND-NOD**	**NOND-OD**	**OND-OD**	**NOD-OD**
Adiponectin-leptin	−0.59⁣^∗^	—	−0.77⁣^∗∗∗^	—	−0.62⁣^∗∗^
Adiponectin-cholesterol	—	—	−0.56⁣^∗∗^	—	—
Adiponectin-HDL	0.79⁣^∗∗∗^	—	0.77⁣^∗∗∗^	—	0.60⁣^∗∗^
Adiponectin-TG	—	—	−0.70⁣^∗∗∗^	0.52⁣^∗^	—
Adiponectin-insulin	—	—	−0.73⁣^∗∗∗^	−0.58⁣^∗^	−0.54⁣^∗^
Adiponectin-RANTES	—	—	−0.70⁣^∗^	—	—
Adiponectin-ENA78	—	0.71⁣^∗^	0.70⁣^∗∗^	—	—
Adiponectin-eotaxin	—	0.71⁣^∗^	—	—	—
Adiponectin-IL13	—	—	—	—	−0.96⁣^∗∗∗^
Adiponectin-IL2	—	—	−0.71⁣^∗^	—	—
Adiponectin-IL10	−0.82⁣^∗∗^	−0.7⁣^∗^	−0.75⁣^∗∗^	—	—
Adiponectin-IL22	−0.88⁣^∗^	—	−0.95⁣^∗∗∗^	0.79⁣^∗^	0.83⁣^∗^
Adiponectin-IL23	—	—	—	—	—
Adiponectin-IL27	−0.93⁣^∗∗^	−0.88⁣^∗∗^	−0.78⁣^∗∗^	—	—
Adiponectin-IP10	—	—	—	—	−0.65⁣^∗^
Adiponectin-MCP3	0.68⁣^∗^	—	—	−0.72⁣^∗^	—
Adiponectin-MIP1*α*	—	—	—	—	—
Leptin-cholesterol	—	—	0.67⁣^∗∗∗^	—	—
Leptin-HDL	−0.57⁣^∗^	—	−0.57⁣^∗∗^	—	—
Leptin-TG	0.60⁣^∗^	—	0.84⁣^∗∗∗^	—	—
Leptin-insulin	—	—	0.64⁣^∗∗∗^	—	—
Leptin-ENA78	—	—	—	—	—
Leptin-eotaxin	—	—	—	0.67⁣^∗∗^	0.85⁣^∗∗∗^
Leptin-GCSF	—	—	—	—	—
Leptin-IFN*γ*	—	—	—	—	—
Leptin-IL13	—	—	—	—	0.79⁣^∗^
Leptin-IL2	—	—	—	—	0.81⁣^∗^
Leptin-IL23	—	—	—	—	—
Leptin-IL6	—	—	—	—	—
Leptin-IP10	—	—	—	—	0.72⁣^∗^
Leptin-MCP3	—	—	—	—	—
Leptin-MIP1*α*	—	—	—	—	—
Leptin-TNF*α*	—	—	—	—	—

*Note:* “—” means no correlation was found.

⁣^∗^*p* < 0.05.

⁣^∗∗^*p* < 0.01.

⁣^∗∗∗^*p* < 0.001.

## Data Availability

The data used to support the findings of this study are included in the article. The datasets analyzed during the current study are available from the corresponding author upon reasonable request.
